# Effect of vitamin A on recovery from the acute phase of multiple sclerosis-related optic neuritis, double-blind, randomized, placebo-controlled trial

**DOI:** 10.22088/cjim.14.1.23

**Published:** 2023

**Authors:** Shiva Rahimi-Dehgolan, Maryam Masoudi, Shahram Rahimi-Dehgolan, Amir Reza Azimi, Mohammad Ali Sahraian, Seyed Mohammad Baghbanian, Abdorreza Naser Moghadasi

**Affiliations:** 1 Multiple Sclerosis Research Center, Neuroscience Institute, Tehran University of Medical sciences, Tehran, Iran; 2 Physical Medicine and Rehabilitation Department, Imam Khomeini Hospital Complex, Tehran University of Medical Sciences, Tehran, Iran; 3 Department of Neurology, Faculty of Medicine, Mazandaran University of Medical Sciences, Sari, Iran

**Keywords:** Multiple sclerosis, Optic neuritis, Vitamin A, Axonal damage

## Abstract

**Background:**

Optic neuritis (ON) is one of the main neuro-ophthalmic presentations of multiple sclerosis (MS), and it causes optic nerve atrophy and axonal loss. However, so far, there is no effective treatment to improve long-term outcomes.

**Methods:**

In a double-blind placebo-controlled randomized clinical trial, 50 patients with MS-related ON were allocated into two arms (24 in the control group and 26 in the intervention group) receiving either 25000IU retinyl palmitate or an identical placebo for six months. Visual evoked potential (VEP), visual acuity, and the retinal nerve fiber layer (RNFL) thickness were evaluated and compared before and after the treatment.

**Results:**

RNFL thickness reduction in the affected eyes at sixth month compared to the baseline were 14.81 and 19.46 μm, in the intervention and control groups, respectively (P=0.017). However, VitA therapy did not affect visual acuity and VEP.

**Conclusion:**

Vitamin A supplementation in the patients with acute ON in MS could lessen optic nerve axonal loss.

Demyelinating acute optic neuritis (ON) is the inflammation of the optic nerve mostly presented as a unilateral acute visual loss with retrobulbar pain exacerbated with eye movement. The most common underlying cause of ON is multiple sclerosis (MS), as around half of people with ON would eventually develop MS (1), and up to 70% of patients with MS experience ON at least once in their disease course (2). However, ON may have other important causes including neuromyelitis optica spectrum disorder (NMOSD), myelin oligodendrocyte glycoprotein (MOG)-IgG, systemic vasculitis, sarcoidosis. ON related to these disorders are different from MS in term of pathogenesis, severity and prognosis. Patients with NMOSD can suffer from severe and bilateral visual loss. So, early treatment of ON is necessary in these patients. Visual recovery in MS-related ON is favorable, and more than 90% of patients have visual acuity better than 20/40(2); however, ON would lead to optic nerve atrophy despite normal vision, which is suggested to be progressive in MS patients (3,4). Like many other autoimmune diseases, the standard care of ON includes intravenous injection of corticosteroid (5), which through different trials, did not offer any benefit in long-term visual acuity and is suggested to only hasten the short-term recovery. Different agents have been studied as a candidate in ON and MS-related ON treatment in last decades, yet no drug has been approved (6). One of the aspects that gained attention in MS and ON treatment is diet modification and supplement therapy (7). 

Observational studies in MS patients showed reduced serum levels of minerals and vitamins, especially fat-soluble vitamins, namely vitamin D and A (7–9). Vitamin A (vit A, retinol) and its derivatives are currently under investigation for their beneficial role in autoimmunity. Vit A has a crucial role both in cellular differentiation and immune system development and hemostasis. Evidence shows that vit A is essential for the maturation of the immune system and promotes both cellular and humoral immunity. In addition, it regulates the immune system by controlling the regulatory T cell (Th) and inhibiting autoimmunity process by suppressing Th1 and Th17 cells (10). Moreover, retinoid signaling takes part in many aspects of the nervous system development, notably myelination and axonal regeneration (11). 

 The bulk of evidence backing potential beneficial role of retinol in MS is mostly indebted to animal and in vitro studies, while related human studies in this domain are infrequent. Nevertheless, in several studies, the effect of vit A was evaluated in MS patients, which showed controversial results. In a study by Yokoto et al. (2017) on 23 MS patients, the low serum retinol level was related to brain atrophy through years, directly (12). In another longitudinal study on 88 relapsing-remitting MS patients, the increment in the serum retinol level improves the MRI outcome in term of new T2 lesions and Gadolinium enhancement plaques (13). While in a randomized control trial by Bitarafan et al. in 2015, one-year supplement therapy with vit A in 101 MS patients did not affect the MRI lesions and relapse rate, although it improved their multiple sclerosis functional composite (MSFC) score (14). In addition, Bitarafan et al. found that vit A therapy alleviates depressive symptoms and fatigue in MS patients (15). 

Considering the possible role of vit A in modulating autoimmune demyelinating syndromes like MS and as there is no efficient therapy for MS-related ON as part of these syndromes, in this study, we sought the possible effects of adjuvant vitamin A supplement therapy on both MS-related ON functional outcome (visual acuity) and its six-month neuroaxonal damage.

## Methods

**Study design: **This randomized identical placebo-controlled blind trial was conducted in a single tertiary center to investigate the effect of supplement therapy with vit A on the recovery time and course of ON. All participants were referred to our center from March 2015 to February 2016 with the confirmed diagnosis of ON. The present research complied with the Declaration of Helsinki 1975 and was approved by the National Committee for Ethics in Biomedical Research at Tehran University of Medical Sciences (ethical code: IR.TUMS.MEDICINE.REC.1395.1284). The aim and setting of the study were explained to all the patients. They were all informed that participating is voluntary and denying it would not affect their treatment. All participants provided written informed consent before entering the study, and all patients received the standard care for ON.

**Participants: **Participants aged 18 to 55 years old referred to our center with first episode of acute unilateral optic neuritis in the context of previously diagnosed MS (16) or as a high-risk Clinically Isolated Syndrome (CIS)(17). The high-risk CIS were CIS with silent MS-compatible MRI lesions or CSF oligoclonal bands (OCBs). The extent of vision loss in the patients should have been 6/10 or lower (16), and the subject should not have received corticosteroid in their recent ON before enrollment. Exclusion criteria were the following: the history of other neurologic diseases, especially neuromyelitis optica spectrum disorder (NMOSD), and the presence of any eye pathologies besides AON diagnosed via an ophthalmologist’s examination. Moreover, patients with contraindication of vit A use like pregnancy or prior liver problems, as well as previous history of increased intracranial pressure or concurrent autoimmune diseases, were excluded from the study. All included participants should be enrolled in the study within two weeks from their attack. The diagnosis of AON was set by an experienced ophthalmologist based on the clinical presentation, physical examination (fundoscopy), or other diagnostic procedures such as magnetic resonance imaging (MRI). Lumbar puncture and cerebrospinal fluid (CSF) analysis were done per-requisite. Snellen charts were used for determining the visual acuity. Visual acuity of 6/10 is frequently described as eyesight of seeing details from six meters away compared to normal eyesight, which sees the same details from ten meters

**Setting & intervention: **The participants were randomly assigned with a 1:1 ratio to vitamin A and identical placebo regimen arms. Allocation sequences were generated by a computerized randomization technique using permuted blocks of 4. Participants, physicians, technicians, and outcome assessors were all unaware of the assigned regimen. At baseline for each patient, demographic data, past medical history, and drug history were gathered. Moreover, the visual acuity was double-checked, and the retinal neural fiber layer (RNFL) thickness was assessed using optical coherence tomography (OCT) in both eyes to measure the reduction of the thickness of the affected eye nerve compared to the non-affected eye. In addition, the rate of latency was examined by visual evoked potential (VEP) in both eyes. 

 All participants received the standard care for two weeks containing intravenous methyl prednisolone 1 g/day for five days followed by oral prednisolone 50 mg tapered in 9 days. The patients who required additional clinical interventions such as plasmapheresis in their treatment course were excluded from the analysis. After completing the two-week treatment course of ON, in the intervention arm, patients were prescribed to take oral form of vitamin A supplement (25000IU retinyl palmitate daily (Zahravi Pharmaceutical Co., Iran)) for six months, while the patients in the control group received an identical placebo. All participants were alerted about the possible side effects of vitamin A. 

**Follow-up and outcomes: **The follow-up period in this study was six months, through which all patients were evaluated for the elevation of liver enzymes every two months. Adverse side effects of retinol consumption in all participants, especially increased intracranial pressure signs and symptoms, were routinely monitored via in-person visits or phone calls. After six months, the patients were re-examined for papilledema, visual acuity, and both eyes were also re-examined for RNFL thickness by OCT and VEP with the same protocol and previously mentioned devices. The primary outcomes in this study were changes in RNFL thickness, P100 latency in VEP, and visual acuity. 

**Imaging protocol: **The latency of p100 component of VEP signal was measured using electroencephalography as one the main outcome and as for VEP imaging protocol the typical pattern reversal VEPs were used. in this method the visual stimulus, a high contrast black-and-white checkerboard which spans 20˚–30˚ in the center of the visual field were presented to patients’ eye while the black and white squares exchange places periodically. The averaged monocular full-field response signal to this reversal was recorded by three occipital electrodes as final VEP result (17). All employed protocols in this study have been consistent with the International Society for Clinical Electrophysiology of Vision (ISCEV) standards (18). The Medelec device was used for the measurement of VEPs. OCT is a technique for measuring RNFL thickness as an indicator of axonal damage. While through measuring the optic nerve, the atrophy of the axon cannot be discerned from myelin thinning, in the RNFL region, the axons of retinal ganglion cells are devoid of myelin, and the entire volume of tissue is made up of axons which make it a perfect area for investigating axonal degeneration.

The employed OCT techniques in our study were a combination of time-domain and spectral-domain OCT. In time-domain OCT, super luminescent diode (810 nm) with single-photon detector and moving mirror was used for image acquisition (ranging from vitreoretinal interface to retinal pigment epithelium) with a scanning speed of 400 A-scans per second. Here, the axial and transverse resolution were 10 µm and 20 µm, respectively. As for spectral-domain OCT, through applying enhanceddepth imaging mode, the depth of posterior cortical vitreous to sclera was imaged. In this protocol, broadband super-luminescent diode source (840 nm), array of detectors and fixed mirror were employed. The scanning speed was 27,00070,000 A-scans per second with an axial resolution of 57 µm and transverse resolution of 14‑20 µm (19). The quality parameters for the images were as follows: SNR = 40, SS = 8, IR = 116.32, TSR = 0.344, and QI = 40.0(19). CIRRUS HD-OCT 5000 was used for this study. 

**Data Analysis: **The sample size was calculated using the software: G*power version 3.1.9.7 (20). For each group, the sample size of 24 patients was determined to detect the clinically significant mean difference of 5 μm in RNFL(19) thickness given to provide a power of 80% for a type two error of 0.05 with an estimated drop out 50%.

 The data were analyzed using the software SPSS Version 22.0 (Armonk, NY: IBM Corp.). Demographic and clinical data were summarized as frequency (percentage, for categorical variables) mean +standard deviation (SD), for continuous variables, or median (interquartile range for ordinal variables). Quantitative variables (VEP and OCT) were compared between groups at baseline and after six months using independent student t-test, while for qualitative variables, chi^2^ test was used; for assessing the association between continuous variables, quantitative correlation coefficient was computed. All *p *values were measured for two-sided comparison with a significance level below 0.05. While required, for multivariate analysis and confounding variable control, logistic regression and linear regression tests were used.

## Results

Fifty-two patients were eligible to enroll in this study; out of them, two individuals were excluded from the analysis (both from the placebo group, one of them received plasmapheresis in the follow-up time, and the other refused to continue the study). Finally, the data of 50 patients were analyzed (40 women and ten men; 24 in the control group and 26 in the intervention group). The mean age of all the patients was 29.60 years old (20-47). The mean MS duration was 9.52 (0-36) months. Moreover, this episode of ON was the first attack in 20 patients. The baseline characteristics were well-balanced in two groups summarized in table 1. All patients arm tolerated the daily dose of vit A, and no patients showed severe side effects and LFT elevation. 

The mean visual acuity in all enrolled patients before and after the six-month treatment was 4.86 and 9.44, respectively. Comparing separately in either intervention or control group, the mean of visual acuity before and after the treatment was 4.69 and 9.54, versus 5.04 and 9.33, respectively, showing no significant change in vit A group compared to control (table 2). While both OCT and VEP results of fellow eyes in both in vit A and control arms showed no difference before and after treatment, OCT evaluation of affected eye in the vit A group demonstrated that RNFL thickness decreased by 14.81 μm on average while this difference was around 19.46 μm, in the control group (P=0.017) through 6 months. The VEP study in the affected eye showed that the mean P100 latency in the intervention arm was 109 ms before and 122.25 ms after the treatment. However, it was 108.9 ms and 122.04 ms in the control group, indicating no significant difference compared to vit A group. We did not find any association between the gender, age, duration of disease, and our main outcomes (table 2).

**Table 1:** Baseline demographic and clinical characteristics

	**Total** **N=50**	**Vitamin A** **N=26**	**Control** **N=24**	**P-value**
Gender (Female: male)	40:10	22:4	18:6	0.396
Age (Mean+SD), year,	29.6+6.41	29.54+6.33	29.67+6.64	0.946
MS duration (mean+SD), month	9.52+10.6	10.65+11.21	8.29+9.98	0.435
Maintenance therapy:				0.488
Glatiramer acetate (GA)	17	10	7	
Beta interferon	33	16	17	
EDSS:				0.337
1	46	23	23	
2	4	3	1	
Plaque count:				0.423
0	20	10	10	
1	20	9	11	
2	10	7	3	
Previously diagnosed with MS:				0.419
yes	30	17	13	
no	20	9	11	

**Table 2:** Visual acuity, VEP, and RNFL thickness in both groups at the start and 6 months after

	**total**		**control**		**case**		**P- value**
	**Mean**	**SD**	**Mean**	**SD**	**Mean**	**SD**	
AON Onset to treatment(day)	12.94	3.44	12.83	3.29	13.04	3.63	0.835
Visual acuity, after	9.44	0.97	9.33	1.09	9.54	0.86	0.466
Visual acuity before	4.86	1.95	5.04	1.73	4.69	2.15	0.528
∆RNFL µm	17.04	6.99	19.46	7.07	14.81	6.24	0.018
VEP (P100 latency), after, ms	122.14	7.41	122.25	6.99	122.04	7.91	0.92
VEP (P100 latency), before, ms	108.96	5.91	109	6.8	108.92	5.08	0.964

**Figure 1:**
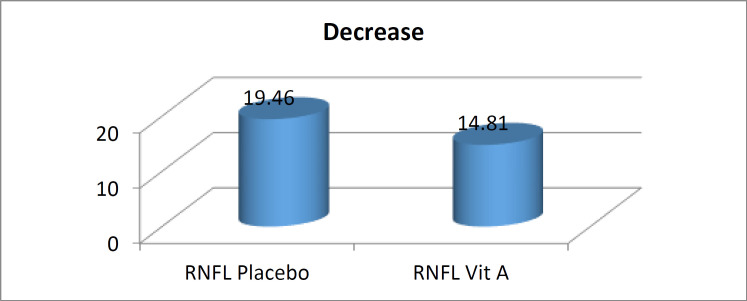
The thickness of RNFL in the affected eye at month 6 compared to the healthy eye at the start in the intervention and control groups

## Discussion

This trial revealed that in MS-related ON, the extent of optic nerve axonal loss was significantly lower in the vit A treated group than the standard care, which proposes that vit A could serve as a neuroprotective agent in demyelinating neuroinflammation like MS-related ON. However, in this study, vit A consumption did not improve the overall functional integrity of the optic pathway. 

Supporting our result, Hiroshi et al. (21) demonstrated that the use of all-trans retinoic acid (ATRA) and a synthetic retinoic acid (RA) receptor agonist for 20 days could ameliorate both clinical and histopathological outcomes in the rat ON model. Moreover, in 2020, the result of phase 2 clinical trial of retinoid acid X receptor (RXR) gamma agonists in MS patients suggested that targeting retinoic signaling could ameliorate myelination in the old lesion and lessen the VEP latency in eyes with electrophysiologic evidence of optic neuropathy (22).

 There are several hypotheses for the justifying observed retinol neuroprotective action in this study. First of all, vit A is known for its immune-modulatory effect in autoimmune disorders, notably MS (11); in an in vitro study on the MS patient-derived B cells, RA-treated cells showed enhanced secretion of interleukin10 (IL10, an anti-inflammatory cytokine) (23) as well in another in vitro study ATRA administration suppressed interleukin17 (IL17) gene expression (24). Although there is no human study on the effect of retinol derivative therapy in the acute phase of MS, in rats with experimental autoimmune encephalomyelitis as an animal model of MS, supplement therapy with vit A metabolites, namely ATRA showed reduced expression of T-bet, IFN-γ, increased level of GATA3 and IL-4 gene expression, and down modulated TH17 differentiation leading to accelerated recovery and better clinical outcome (24). Accordingly, diminished axonal loss observed in our study could be justified with the auxiliary effect of vit A in the early suppression of the autoinflammatory process in the acute phase of disease. 

Furthermore, vit A could alleviate the damage in the acute autoimmune inflammatory phase of attack by reducing oxidative radicals. Evidence proposes that inflammation-induced oxidative stress is accountable partly for the neurodegeneration in MS patients (25). In addition, imbalance in tissue antioxidant capacity could trigger inflammatory processes and predispose MS patients to relapse (25). However, evidence backing the use of antioxidants is still lacking. In 2013, in an animal model of multiple sclerosis, Morvaridi et al. showed that ATRA treatment alleviated the clinical signs of experimental encephalitis and found that induced nitric oxide production was significantly lower in ATRA-treated mouse group (26). Additionally, vit A is assumed to be mandatory for nervous system development and repair, Particularly the importance of retinoic acid receptor β (RARβ) signaling in adult CNS regeneration is illustrated through many studies (27–29). Gancalves et al. in 2018 demonstrated that upregulation RA synthesis by RARβ activation in axonal injury model has a path-determining effect for neural outgrowth (28). Likewise, treatment with RARβ agonist in spinal cord injury induced axonal regeneration and promote functional capability (27). However, the neuroprotective and regenerative effect of RA signaling is mostly studied in neurodegenerative, traumatic and ischemic diseases (29,30) and the role of vit A in compromising axonal degeneration in MS is yet to be found out. Besides, demyelination is determined as a contributor to axonal degeneration in MS; thus, reinforcing the remyelination process after an attack could lessen the axonal loss (32). Retinoic signaling plays a vital role in myelination both postnatally and in adulthood (33,34). Literature suggests that RA increases myelin debris phagocytosis by macrophage, which is essential for remyelination (35). Moreover, a growing body of evidence demonstrated that RARβ, RARα, and retinoid X receptors are important regulators of oligodendrocyte differentiation (34,36). In concordance, in an in vitro study on demyelinated spinal cord of rats, treatment with 9-cis-RA stimulated remyelination (34) and in a rat focal white matter lesion model, RA-loaded lipid nano-capsules promoted oligodendrogenesis in lesion area (37). However, the stimulating effect of vit A metabolites in remyelination seems to be time-dependent. Young Kim et al. in 2017 investigated the effect of different vit A derivatives on oligodendrocyte precursor cells (OPC) differentiation. They found that prolonged treatment with different isoforms could upregulate OPC inhibitors (38). In this study, all isoforms of RA promoted myelination after a week of therapy but through the 27^th^ day showed controversial results, and by 47^th^ day, all suppressed the myelination in vitro. Consequently, one of the plausible reasons for no improvement in VEP and vision of our treatment group could be prolonged treatment with vit A; hence, determining the optimal duration of vit A therapy in future studies is crucial to exploit vit A in MS. 

### Strengths and Limitations:

This study was the first trial investigating the efficacy of vit A supplement therapy in the acute phase of MS-related ON, and has the advantage of adequate sample size with good statistical power and long follow-up. Nevertheless, it faced several shortcomings. Considering that the vit A deficiency is likely to be more abundant in MS patients and as in this study, the baseline and follow-up serum level of vit A were not assessed in patients, the possible interaction of vit A deficiency on treatment response could not be dismissed. However, as the patients were randomized and well-balanced in both groups, this matter is unlikely to disturb our result consistency. Besides, in this study, we had only one follow-up point for the main outcome. Also, short-time outcomes and the course of recovery were not evaluated. Furthermore, this trial only targeted patients with ON, and the benefit of vit A in other forms of MS attacks is currently obscure; thus, future studies are demanded to better investigate the vit A therapy efficacy and mechanism of action in demyelinating diseases. 

### In conclusion:

This paper revealed that vit A supplement therapy is a safe and tolerable candidate for adjuvant therapy in the acute phase of MS-related ON and may mitigate optic nerve atrophy and could lessen optic nerve axonal loss after an ON episode which demonstrated vit A could possess a neuroprotective effect in CNS autoimmune demyelination diseases.
